# Exogenous Addition of Arachidonic Acid to the Culture Media Enhances the Functionality of Dendritic Cells for Their Possible Use in Cancer Immunotherapy

**DOI:** 10.1371/journal.pone.0111759

**Published:** 2014-11-04

**Authors:** Jeetendra Kumar, Rupali Gurav, Vaijayanti Kale, Lalita Limaye

**Affiliations:** Stem Cell Lab., National Centre for Cell Science, Ganeshkhind, Pune, India; Leiden University Medical Center, Netherlands

## Abstract

The development of dendritic cell based vaccines is a promising approach in cancer immunotherapy. For their successful use in the clinics, the propagation and functionality of DCs is crucial. We earlier established a two-step method for the large scale generation of DCs from umbilical cord blood derived MNCs/CD34+ cells. This work aims at improving their functionality based on the following observations: *in vitro* generated DCs can be less efficient in migration and other functional activities due to lower eicosanoid levels. The production of eicosanoids from Arachidonic Acid (AA) can be hampered due to suppression of the enzyme phospholipase A2 by IL-4, an essential cytokine required for the differentiation of DCs. We hypothesized that exogenous addition of AA to the culture media during DC generation may result in DCs with improved functionality. DCs were generated with and without AA. The two DC sets were compared by phenotypic analysis, morphology and functional assays like antigen uptake, MLR, CTL assay and *in vitro* and *in vivo* migration. Though there were no differences between the two types of DCs in terms of morphology, phenotype and antigen uptake, AA^+^ DCs exhibited an enhanced *in vitro* and *in vivo* migration, T cell stimulatory capacity, CTL activity and significantly higher transcript levels of COX-2. AA^+^ DCs also show a favorable Th1 cytokine profile than AA^-^ DCs. Thus addition of AA to the culture media is skewing the DCs towards the secretion of more IL-12 and less of IL-10 along with the restoration of eicosanoids levels in a COX-2 mediated pathway thereby enhancing the functionality of these cells to be used as a potent cellular vaccine. Taken together, these findings will be helpful in the better contriving of DC based vaccines for cancer immunotherapy.

## Introduction

Dendritic cells (DCs) are most efficient antigen presenting cells (APCs) which recognize the universe of antigens and control various types of responses [1], [2]. DCs are capable of capturing antigens, processing them, and presenting them with appropriate costimulation molecules and initiate immune response [3], [4]. DCs are not only critical for the induction of both primary and secondary T and B cell mediated immune responses, but are also important for the induction of immunological tolerance. DCs are at center of the immune system and modulation of the immune response is important in therapeutic immunity against cancer [5]. The unique ability of DCs in antigen presentation and regulation of immune response has made them an attractive adjuvant in cancer immunotherapy [6]. Advances in the *in vitro* DC generation protocols and better understanding of DC biology have resulted in their use as DC vaccines in the clinics. Since its first report in 1995, large numbers of clinical trials have been carried out to evaluate DC-based vaccines against more than a dozen different types of tumours [7], [8], [9]. Clinical use of DCs requires repeated vaccination to induce relatively high frequencies of tumor antigen specific Cytotoxic T lymphocytes (CTLs) and a complete response. This in turn requires a large number of DCs, generated *ex vivo* [10].

DCs can be generated from CD34^+^ cells using a one step or a two step culture systems. In the first type of culture system CD34^+^ cells are grown with a combination of growth factors, where in they are directly differentiated as dendritic cells [11], [12], [13]. On the other hand, in the two steps culture system CD34^+^ cells are first expanded on a large scale as DC precursors. These expanded cells are further differentiated as DCs [14], [15], [16], [17]. Despite the full understanding of the complex DC-mediated regulation of host leukocyte responses, *in vitro* generated DCs may not represent the equivalent of migratory DC *in vivo*, thereby limiting their use as magic bullets to improve the precision and effectiveness in cancer immunotherapy. Recent experimental evidence demonstrate that human monocyte-derived DC (MoDC) may be hampered in their ability to migrate in response to inflammatory as well as homeostatic chemotaxins [18]. Previous reports show that IL-4, which is an important cytokine for *in vitro* DC generation, inhibits many of the downstream pathways of Arachidonic Acid (AA) metabolism resulting in the impaired production of eicosanoids and platelet activating factor (PAF). Prostaglandin E_2_ (PGE_2_) is a member of the eicosanoid family of oxygenated AA derivatives. The first step of PGE_2_ biosynthesis is the release of AA from membrane phospholipids by phospholipases such as phospholipase A2 (PLA_2_). Since eicosanoids and PAF are known to play an important role in processes such as leukocyte migration, natural killer cell activation, and type 2 T helper cell differentiations, the deficiency in biosynthesis of these factors may be responsible for the observed handicaps of MoDCs [19].

We earlier established a two-step plastic adherence method for the large scale generation of DCs derived from both umbilical cord blood CD34+ cells [17] and MNCs (Mononuclear cells) [20]. The DCs generated by our method have a mature phenotype and are functionally active. However one of the cytokines used to generate DCs by our method is IL-4 and as mentioned above IL-4 may affect release of arachidonic acid from the membrane.We hypothesized that exogenous addition of AA to our cultures during the differentiation step may help in further improving the functions of DCs. The rationale for adding exogenous AA was that it may get converted into prostaglandins in a Cyclooxygenases-1 (COX-1) and Cyclooxygenases-2 (COX-2) dependent manner. To check this hypothesis, in the present study we tested the effect of AA addition on *in vitro* DC generation. Our data demonstrated that indeed AA^+^ DCs are superior in functions such as enhanced *in vitro* and *in vivo* migration, T cell stimulatory capacity, antigen uptake, CTL activity, significantly higher transcript levels of COX-2 and a favorable Th1 cytokine than AA^-^ DCs. Thus our findings take us one step closer towards generating the ameliorate form of DC based cancer vaccine.

## Materials and Methods

### Cytokines

The recombinant human cytokines used for the study were Fms like tyrosine kinase 3 ligand (Flt-3L), Thrombopoietin (TPO), Stem Cell Factor (SCF), Interleukin-4 (IL-4), Granulocyte monocyte -colony stimulating factor (GM-CSF), Tumor necrosis factor-α (TNF-α), CD40 Ligand and Chemokine ligand-19 (CCL-19). All recombinant human cytokines were purchased from Peprotech Asia, Revohot Israel.

### Antibodies

The antibodies used for flow cytometry were mouse anti human mAbs: CD1a, CD34, CD11c, CD40, CD8 -APC tagged; CD14, CD20, CD33, CD58, CD80, CD83, CD86, HLA-A2, CD45RA -PE tagged; CD3, CD54, HLA-DR, HLA-ABC -FITC tagged, anti murine CD 45.1 -PB tagged and CCR-7 (purified antihuman rat mAb). All monoclonal antibodies and respective isotype controls used for the study were purchased from BD pharmingen (San Diego, CA).

### Other reagents used

Arachidonic Acid- (Cat. No. is A3555) from Sigma Aldrich, a tissue culture tested product derived from porcine liver. The purity of the product was ≥99% as analyzed by capillary Gas Chromatography.

Ficoll Hypaque-Histopaque (density 1.007 g/ml); Fluorescein isothiocyanate (FITC)-labeled dextran (40 kDa); Wright Stain, Giemsa Stain, Lipopolysaccharide, DMEM (Dulbecco's Modified Eagle Medium) and IMDM (Iscove's Modified Dulbecco's Medium) all were from Sigma Aldrich; ELISA kit (BD OptEIA); Heparin from SRL Pvt. Limited, Mumbai; Hydroxy Ethyl Starch (HES), Rosette sep for T cell isolation (Stem Cell Technologies, Vancouver, Canada); [^3^H] thymidine (240 GBq/milli mole, BRIT, Navi Mumbai, India); Dimethyl sulfoxide (MP biomedical, Ohio, USA); Dynal kit for mRNA isolation (Dynal ASA, Oslo Norway).

### Collection and Processing of Cord Blood Samples

Cord blood (CB) samples were obtained from the local hospitals according to the institutional review board [Institutional Ethics Committee (IEC) and Institutional Committee for Stem Cell Research (IC-SCRT)] –approved ethical guidelines which are in accordance with the Declaration of Helsinki. A prior informed written consent of the mother was obtained. The consent procedure was approved by IC-SCRT. The hard copies of the signed consent forms are numbered and filed with us. The samples were collected in sterile containers containing preservative-free heparin (40 IU of heparin/ml of blood) in plain IMDM. The samples were brought to the laboratory on ice packs and were processed to isolate mononuclear cells (MNCs).

### Isolation of MNC

Cord Blood MNCs were separated by Ficoll- hypaque gradient centrifugation. The cells at the interphase were collected and washed to remove the Ficoll-hypaque. Nucleated cell count (Crystal violet staining) was taken and the MNCs were used for DC generation.

### Isolation of T-Cells from Peripheral Blood

Peripheral blood was collected from healthy donors according to institutional review board. [Institutional Ethics Committee (IEC) and Institutional Committee for Stem Cell Research (IC-SCRT)] –approved ethical guidelines which are in accordance with the Declaration of Helsinki. A prior informed written consent of the healthy donor was obtained. The consent procedure was approved by IC-SCRT. The hard copies of the signed consent forms are numbered and filed with us. T cells were isolated from blood by using negative selection kit (Rosette Sep) as per the manufacture's instruction (Stem Cell Technologies). After Ficoll- hypaque gradient centrifugation cells at the interphase of Ficoll and medium were collected, washed then used in the experiments.

### *In Vitro* Generation and Culture of DCs

#### Expansion cultures and Plastic adherence

MNCs (fresh or frozen) were expanded for 3 weeks, then enriched for CD14+ cells by plastic adherence and subsequently differentiated as described earlier [17], [20]. Briefly, after 21 days of expansion the cells were washed and seeded at the density of 3×10^5^ cells/well in six-well plate containing 2 ml of IMDM supplemented with 1% autologous plasma. The cells were kept for 1.5 hour at 37°C in 5% CO_2_ incubator and the non-adherent and the loosely adherent cells were removed by washing with IMDM. The adherent cells were used for DC generation.

#### Differentiation of DCs from precursors in presence of AA (AA^+^ DCs) and absence of AA (AA^-^ DCs)

The precursor cell populations were divided into two sets. Cells were induced to differentiate as DCs by culturing them in GM-CSF (50 ng/ml) and IL-4 (30 ng/ml) for 3 days, on day 3^rd^ GM-CSF (50 ng/ml) plus TNF-α (30 ng/ml) were added to cultures and further maintained for 4 days in IMDM supplemented with 5% autologous plasma. In order to assess the effect of AA, one set of culture was maintained with 100 µM of AA in addition to the conditions mentioned above. On day 7, the cells were subjected to maturation with a combination of TNF-α, lipopolysaccharide, and CD40L, at the concentration of 100 ng/ml each, for 48 hours. The experimental design is depicted in the form of a flow chart Fig. S1. In all the experiments DCs generated without AA was considered as control set and with AA was considered as test set.

#### Morphological Analysis: Wright's and Giemsa Staining

The adhered cells in culture plates were fixed in methanol for 10 minutes at room temperature and stained with Wright's and Giemsa Stain. Observations were made under inverted microscope and images were taken (Olympus IX70).

#### Phenotypic Analysis: Flow cytometry

Mature AA^+^ and AA^-^ DCs were washed and suspended at 2×10^5^ cells in 100 µL of cold phosphate-buffered saline (PBS) and staining was carried out as per the described protocol [17], [20]. For CCR-7 staining, cells were incubated with primary followed by secondary and tertiary antibodies for 25 minutes each. Cells were washed twice, after each antibody incubations and finally resuspended in 1% paraformaldehyde. Cells were analyzed on a flow cytometer (FACS Canto II from BD Pharmingen- San Jose, California). Cell debris was eliminated from the analysis using a gate on forward and side scatters, and the total cells were analyzed. Data were analyzed and histogram overlays were prepared using computer software FACS Diva and Cell Quest Pro (Beckon Dickinson San Jose California), respectively.

### Functional Characterization

#### Endocytosis assay with FITC-dextran

Immature DCs harvested on day 5 were incubated with FITC-dextran (20 µg/mL), either at +4°C (internalization control) or at +37°C, for 30 and 60 minutes. The cells were then washed thrice with cold PBS containing 0.01% sodium azide and 1% BSA. Cells were re suspended in 1% paraformaldehyde and were acquired on a flow cytometer (Canto II, BD).

#### Chemotaxis

The chemotaxis of the *in vitro* generated DCs toward CCL-19 was assessed in 24-well cell culture plates with BD Falcon ^™^ Cell culture 0.8 µm inserts. 500 µL of IMDM with or without rhCCL-19 (final concentration, 500 ng/mL) was added to the lower chamber as per the described protocol [17], [20]. To check the specificity of chemokine receptor (CCR) interaction, in some experiments DCs were pretreated with CCR-7 blocking antibody for 1 hr in 1× PBS and then their migration towards CCL -19 were studied as described above. The migrated cells were stained by trypan blue dye and the viable cells were counted using hemocytometer. The results are expressed as the mean number of migrating cells ± standard deviation (SD).

#### Mixed Leukocyte Reaction (MLR)

Allogeneic T cells were distributed at 10^5^ cells per well into round bottom 96-well micro plates (Greiner Bio-One) and were cocultured in the presence of graded numbers of irradiated DCs (2500 rad, Co source) in 200 µL of medium containing 10% pooled cord blood AB+ plasma. Thymidine incorporation was measured on Day 3 after an 18-hour pulse with [^3^H] thymidine (1 µCi/well) by using standard procedures [17], [20].

#### Cytokine measurement

Supernatants from the *in vitro* generated DC cultures were collected at 48 hrs after the addition of maturation stimuli and were kept frozen at −20°C. The supernatants were subsequently assayed for cytokine content (IL-10 and IL-12 p70) by ELISA using an ELISA kit as per the manufacturer's instructions. Amount of interleukins present in the supernatants were calculated by standard curve method.

### *In vitro* CTL (Cytotoxic T lymphocytes) assay

#### Preparation of cell lysate

MCF-7 is a HLA-A2 restricted cell line and was used as the target cell line in the CTL assay. Lysis of MCF-7 cells was done by repeated freeze thawing then followed by sonication. After filter sterilization protein estimation was done and the lysate was stored at -80°C for antigen pulsing to the DCs.

#### Generation of effector cells and the CTL assay

HLA-A2 positive cord blood MNCs were used to generate DCs. Immature DCs were incubated with lysate of MCF-7 at a final concentration of 100 µg/ml of protein along with KLH (Keyhole limpet hemocyanin) 25 µg/ml for 48 hr as maturation stimuli in the culture medium for cross presentation of tumor antigen to autologous naïve T cells. For the control set of DCs, the maturation stimuli comprised of 100 ng/ml each of LPS, CD40L and TNF-α. The autologous naïve T cells were obtained by sorting of CD3, CD8, and CD45RA positive cells on FACS ARIA (BD). The naïve T cells were co cultured with MCF-7 lysate pulsed DCs that were generated from the same UCB sample with or without AA. DC-T cell co culture was maintained for 3 weeks with the addition of cytokines IL-2 (0.1 µg/ml) and IL-7 (5 µg/ml) and weekly re stimulation with fresh antigen pulsed DCs to generate effector Cytotoxic Killer Cells. CTLs thus generated from LPS pulsed and lysate pulsed AA^+^ DCs/AA^-^ DCs were co cultured with the target cells MCF-7 for 18 hours. Cells were then washed with plain media and then pulsed with [3H] thymidine for 8 hours. Percent killing of MCF-7 was calculated by P-JAM assay [21], [22] using the formula- % killing  =  CPM [Target alone – Target + Killer] X 100/CPM Target Alone.

### RT PCR analysis

mRNA was isolated from the AA^+^ DCs and AA^-^ DCs using Dynabeads mRNA DIRECT kit as per the manufacturer's instructions. The eluted mRNA were reverse transcribed to cDNA by using reverse transcriptase (Sigma Aldrich) and oligo-dT primers (Invitrogen) as per the instructions and PCR was performed with specific primers. The thermal cycle used was (95°C for 2 min, 95°C for 45 sec, annealing (as per different genes) for 45 sec, extension 72°C for 2 min) for 35 cycles and a final extension at 72°C for 5 minutes. The primer sequences used are described in Fig. S2.

### Homing of mature DCs in NOD-SCID mice

The NOD/LtSZ-scid/scid mice were obtained from the Jackson Laboratories and were bred in the animal facility of our institute. The study was conducted adhering to the institution's guidelines for animal husbandry and has been approved by IAEC-NCCS/CPCSEA (Institutional animal ethical committee-NCCS/Committee for the Purpose of Control and Supervision of Experiments on Animals. Approval number: IACUC-Institutional Animal Care and Use Committee, EAF-Experimental Animal Facility/2004/B-71). Animal procedures involving intra-venous infusion were carried out under anesthesia. Mice at 4–6 weeks of age were exposed to sub lethal dose of 300 rads total body irradiation from a ^60^Co source (Gamma Chamber 5000, BRIT, Navi Mumbai, India). 10^6^ AA^+^ DCs and AA^-^ DCs were infused through the tail vein into the sub lethally irradiated mice (n = 43; infusion of AA^+^ DCs – 20 mice, AA^-^ DCs – 20 mice and PBS – 3 mice). Mice were sacrificed after 18 hours to assess homing of human DCs in bone marrow. Homed cells were identified as DC by staining with human CD11c mAb. Murine CD 45.1 mAb was used to negate the presence cells of murine origin.

### Statistical analysis

Different variables of AA^+^ DCs and AA^-^ DCs were compared. Statistical analysis was done using Sigma Stat (Version-2.03) and graphs were prepared using Sigma Plot software (Version 10) (Jandel Scientific, San Rafael, CA, USA) and GraphPad Prism 5 Software (San Diego, California, USA). Statistical analyses of differences between the two groups were performed using a t test. Probability value: P≤0.05(*), P≤0.01(**) & P≤0.001(***) were considered statistically significant.

## Results

### Morphological and Phenotypic Characterization of DCs

#### The AA^+^ DCs and AA^-^ DCs show similar morphology

The DC precursors generated after 21 days of expansion of MNCs were enriched by the plastic adherence method and further differentiated as DCs [20]. There was marginal difference in the absolute number of DCs generated by the two methods from same number of starting population (approximately 3×10^7^ AA^+^ DCs and 2.5×10^7^ AA^-^ DC per 10^7^ input MNCs). The morphology of AA^+^ DC and AA^-^ DCs was comparable. It was found that the DCs from both the sets exhibited the characteristic veiled morphology and DC clusters; Fig. S3. Phase contrast images showed typical adherent immature DCs, in both the sets [S-3(A)]. After addition of maturation stimuli typical nonadherent DC clusters were observed in both the cultures [S-3(B)]. Wright-Giemsa staining of mature adherent cells shows the presence of DCs with dendrites in the two sets [S-3(C)].

#### The AA^+^ DCs and AA^-^ DCs show similar DC phenotype

**Figure 1:**
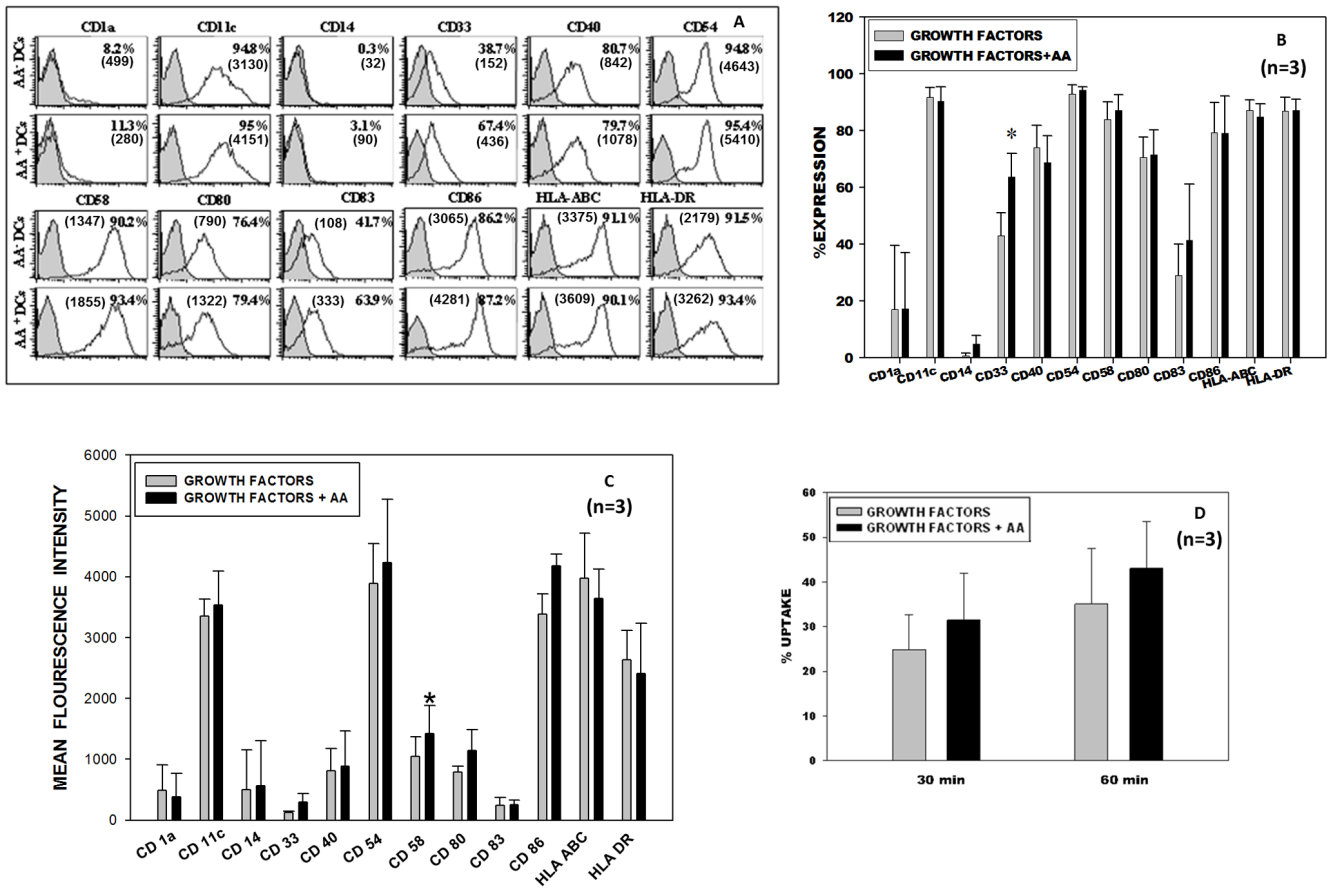
Phenotype and Antigen uptake of AA^+^ DCs and AA^-^ DCs: DCs were generated from the expanded DC precursor cells with and without AA. (A) FACS histogram profile of a representative experiment. Filled histograms show the isotype control and open ones show the specific phenotype with respective MFI values in the brackets. (B) It is seen that fully mature DCs were generated and there was not much difference in the expression levels of different surface markers, except CD33 (myeloid lineage) was higher in AA^+^ DC sets (mean ± SD, n = 3, P≤0.05 *). (C) Bar graph for DC specific and associated markers in terms of MFI, for CD 58 (adhesion molecule) the difference was found significantly higher (mean ± SD, n = 3, P≤0.05 *). (D) Receptor mediated antigen uptake of AA^+^ DCs and AA^-^ DCs: Dextran-FITC uptake by DCs (n = 3, mean ± SD). The control value (uptake at 4°C) is deducted from the respective test value (uptake at 37°C).

*In vitro* generated DCs were stained for a panel of DC specific and DC associated markers and analyzed on the flow cytometer. Fig. 1A depicts the flow cytometric profile of one representative sample with percent positive cells and MFI values in brackets. AA^+^ DCs and AA^-^ DCs showed a high and an equivalent percentage of cells expressing different costimulatory molecules like CD40, CD80, CD86, DC-associated integrin CD11c, adhesion molecules like CD54 and CD58, MHC class II molecules like HLA ABC and HLA DR. Fig. 1B and 1C depicts the phenotypic profile in terms of the percent positivity and MFI respectively, of three samples (mean ± SD, n = 3). The expression of myeloid lineage specific marker CD 33 was found significantly higher on AA^+^ DCs (67.4%) as compared to AA^-^ DCs (38.7%) Fig. 1B. MFI values of adhesion molecule CD 58 was found significantly higher on AA^+^ DCs (1423) as compared to AA^-^ DCs (1048) Fig. 1C. Taken together, AA^+^ DCs and AA^-^ DCs show similar morphology and typical interstitial DC phenotype.

### Functional Characterization of DCs

#### The capability of endocytosis of AA^+^ DCs was higher as compared to AA^-^ DCs

Immature DCs are characterized by the ability of antigen uptake using different mechanisms. We analyzed the ability of the *in vitro* generated DCs for their receptor-mediated antigen uptake by measuring the FITC tagged dextran uptake. AA^+^ DCs and AA^-^ DCs were equally efficient in receptor-mediated uptake at 30 min and 60 min time intervals tested. AA^+^ DCs exhibited high and comparable FITC-dextran uptake as compared to AA^-^ DCs Fig. 1D (mean ± SD, n = 3). *In vitro* generated DCs exhibited more uptake at 60 min interval compared to 30 min, showing that they are functionally active and exhibit an increased uptake with increase in time.

#### Improved *in vitro* chemotaxis of AA^+^ DCs towards CCL19

**Figure 2:**
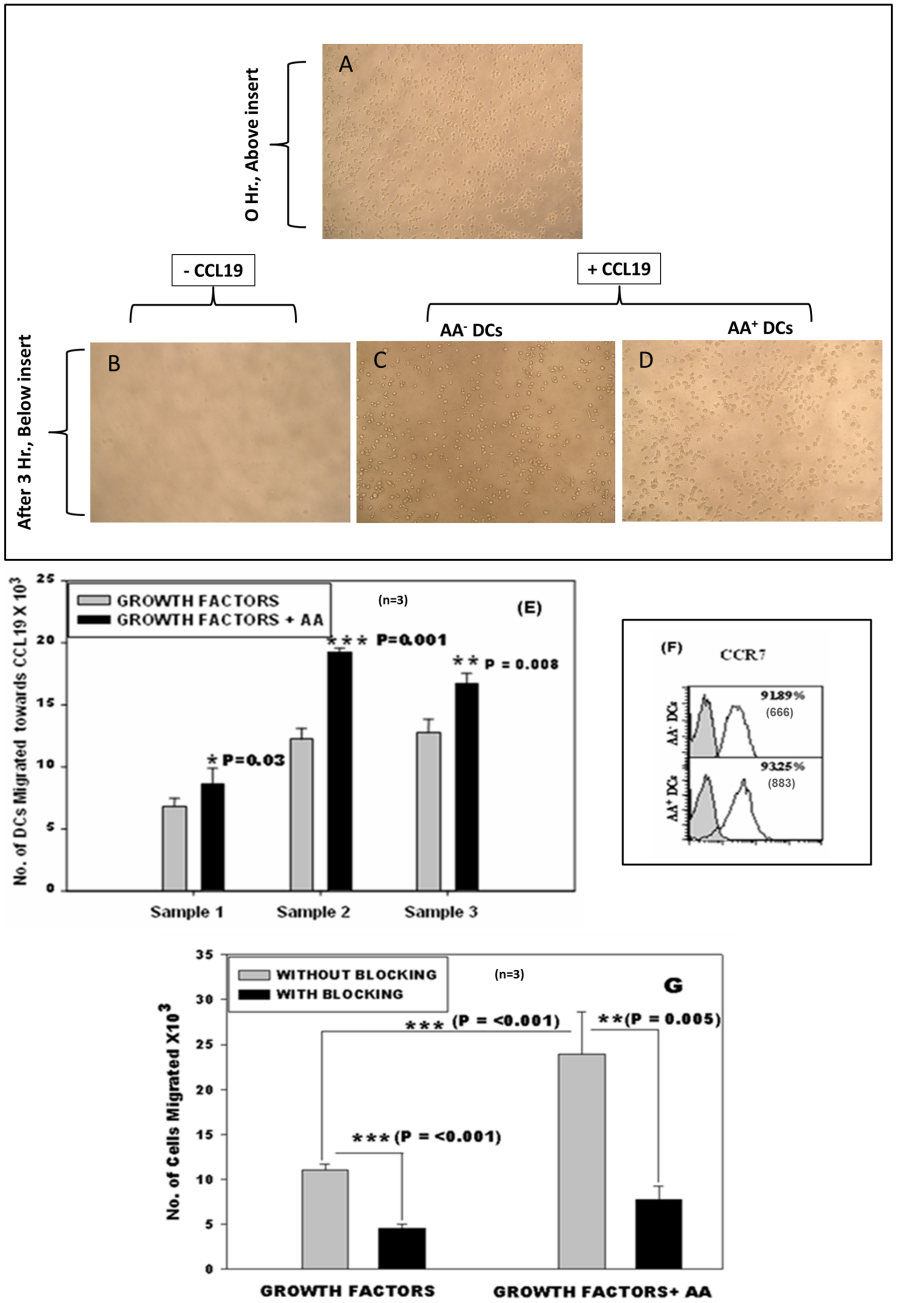
Chemotaxis and CCR7 expression: (A) DCs suspended in upper chamber of well before migration. After 3 hours (B) No spontaneous migration was observed in the wells, where CCL-19 was not added. Less no. of AA^-^ DCs (C) migrated towards CCL-19 than AA^+^ DCs (D). (E) AA^+^ DCs showed statistically efficient migration towards CCL-19 compared to AA^-^ DCs (n = 3, mean ± SD). (F) FACS histogram profile of a representative experiment showing expression of chemokine receptor CCR-7 along with MFI values in brackets. Filled histograms show the isotype control and open ones show the specific phenotype. (G) Blocking with CCR-7 Ab causes significant reduction in migration of both AA^+^ DCs and AA^-^ DCs (n = 3, mean ± SD). [P≤0.05(*), P≤0.01(**) & P≤0.001(***)].

Migration of DCs toward a chemokine gradient is an important functional property because in the immunotherapy protocols they must be capable of migration from the site of injection toward the lymph nodes, where they interact with the T cells and initiate the immune response. Fig. 2A shows, the cells suspended in IMDM added to the upper chamber in well at 0 hrs. No spontaneous migration was observed in the wells after 3 hrs, where CCL-19 was not added (Fig. 2B). More number of AA^+^ DCs (Fig. 2D) migrated towards CCL-19 as compared to AA^-^ DCs (Fig. 2C) and the difference was statistically significant (*p≤0.05, **p≤0.01; n = 3; Fig. 2E). Migration towards CCL19 is mainly because the DCs express CCR7 receptor. When we stained the DCs of the two sets for the CCR-7 receptor antibody, the AA^+^ DCs and AA^-^ DCs showed 93.25% and 91.89% positive cells respectively along with MFI values in brackets (Fig. 2F). Mean MFI values of 3 samples for AA^-^ DCs were 821±156 and AA^+^ DCs were 1089±392. Thus both sets exhibited high and comparable level of CCR7 expression. The specificity of CCR7-CCL19 receptor ligand reaction was further confirmed by blocking the receptor with CCR-7 blocking Ab and then testing the migratory ability. As seen in Fig. 2G there was a significant reduction in migration of cells pretreated with blocking antibody in the two cultures. The migration of the test cells was once again significantly higher than control cells. This data indicated that, the addition of AA during differentiation step of DCs significantly improves their migratory ability.

#### AA^+^ DCs exhibited superior T-Cell stimulatory function

**Figure 3:**
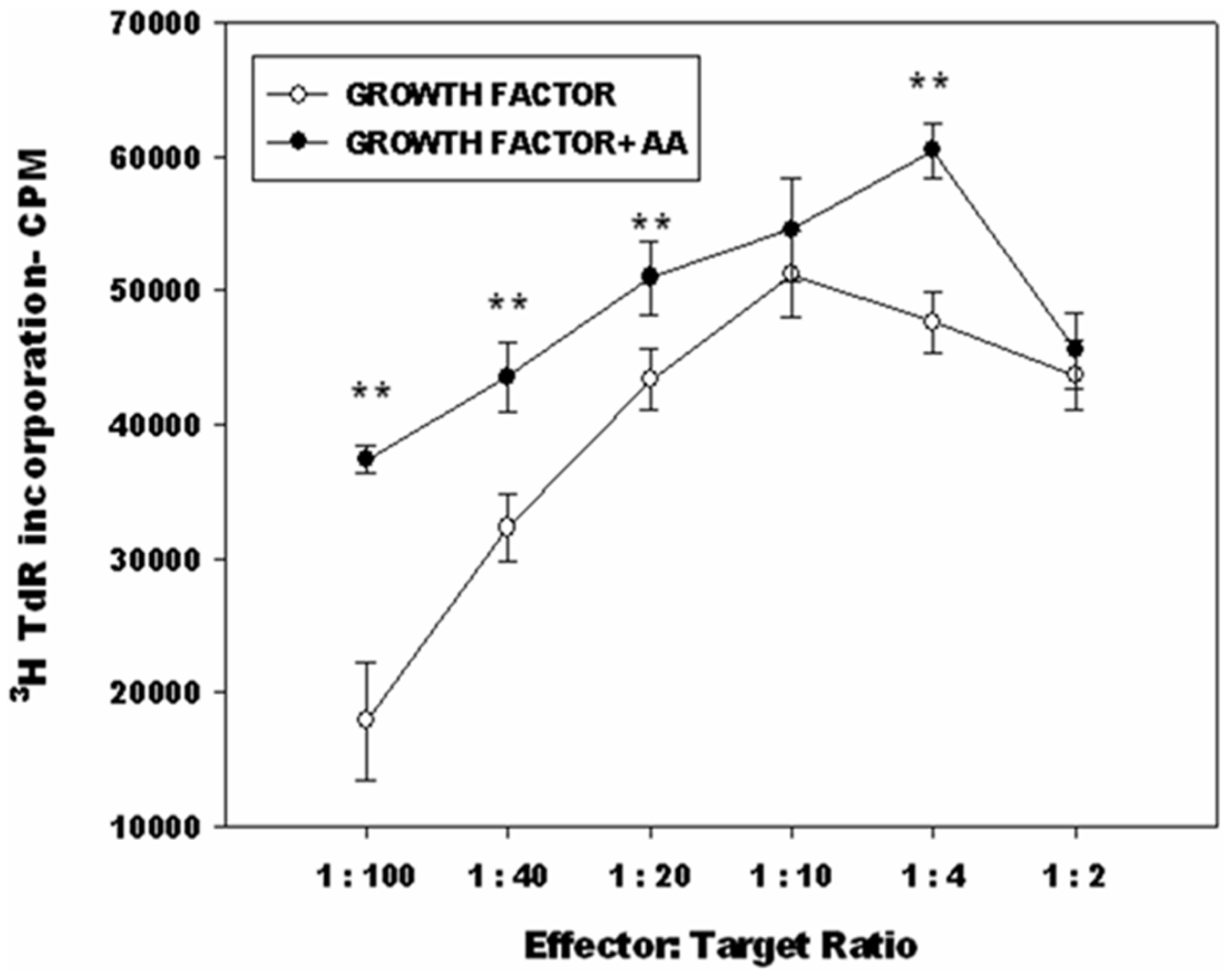
AA^+^ DCs exhibit better MLR than AA^-^ DCs: DCs generated were capable of efficient stimulation of allogeneic T cells. AA^+^ DCs showed better T-cell stimulation than the AA^-^ DC, differences were significant at four out of six DC:T-cell ratios tested (*P≤0.05,**P≤0.01). Representative data from three independent experiments is shown. TdR  =  thymidine.

DCs are characterized by their ability to stimulate allogeneic T cells. This ability was assessed by MLR. The MLR was carried out by mixing the AA^+^ DCs and AA^-^ DCs with allogeneic T cells from peripheral blood in different proportions. Triplicates were taken for each concentration tested. Proliferation of T cells was measured by the thymidine uptake assay as described in methods. Data from Fig. 3 (mean ± SD, n = 3) indicate that the AA^+^ DCs have higher allostimulatory capacity. The differences were statistically significant at four out of six DC: T-cell ratios tested. At 1∶10 ratio, thymidine incorporation in AA^+^ was higher than AA^-^ set.

#### Cytokine Profile of AA+ DCs is more favorable for adoptive immunotherapy

**Figure 4:**
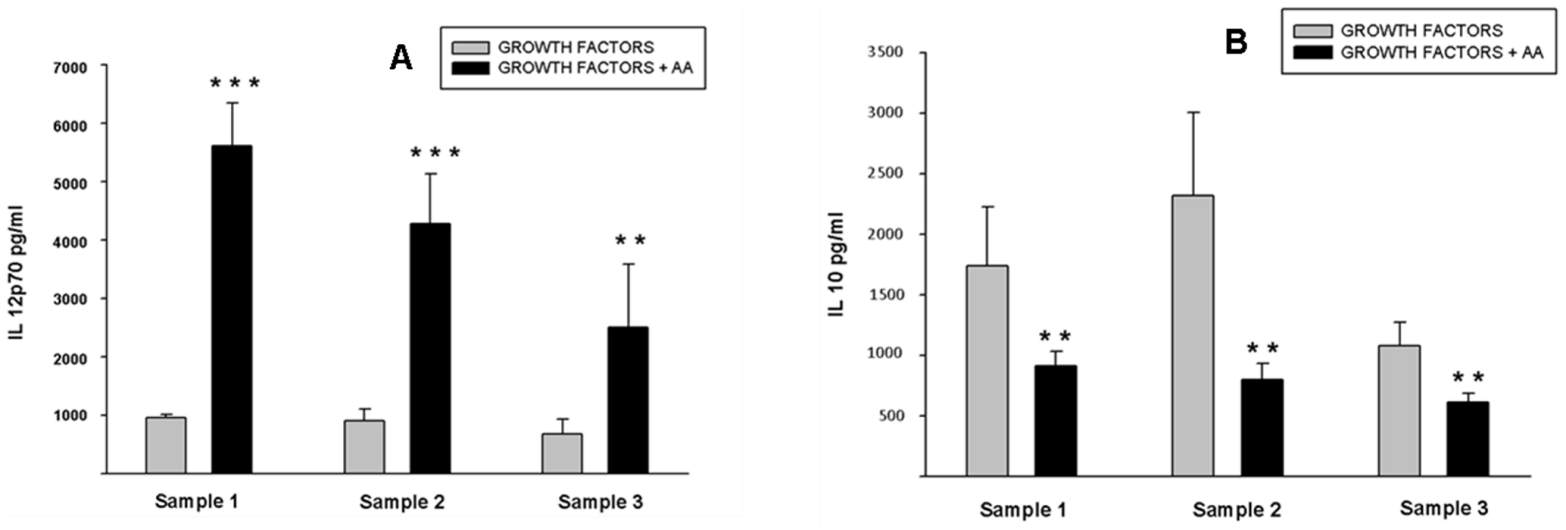
Detection of IL-10 and IL-12 p70 in the culture supernatants of AA^+^ DCs and AA^-^ DCs: *In vitro* generated DCs were assessed for their ability to secrete IL-12 p70 and IL-10. Results show that DCs generated by both culture conditions were capable of secretion of IL-12 p70 and IL-10. (A) AA^+^ DCs showed a better IL-12 p70 secretion compared to AA^-^ DCs. (B) Similarly, AA^+^ DCs have low level of IL-10 secretion profile as compared to AA^-^ DCs. The data of three independent samples are shown (n = 3, mean ± SD). [P≤0.05(*), P≤0.01(**) & P≤0.001(***)].

IL-12 not only signals for development of effector functions in T cells, but also programs the cells to survive as functional memory cells [23]. *In vitro* generated DCs were capable of secreting a high level of IL-12 p70 and a low level of IL-10. The AA^+^ DCs showed a better IL-12 p70 secretion profile as compared to AA^-^ DCs (Fig. 4). AA^-^ DCs on other hand had a higher level of IL-10 secretion compared to that of AA^+^ DCs (n = 3). The ratio of IL12/IL10 was higher in AA^+^ DCs as shown in Fig. S4. These observations suggest that the AA^+^ DCs have a better Th1 favorable cytokine profile as compared to AA^-^ DCs, making them a better choice for clinical application.

### AA^+^ DCs demonstrate higher *in vitro* CTL activity

**Figure 5:**
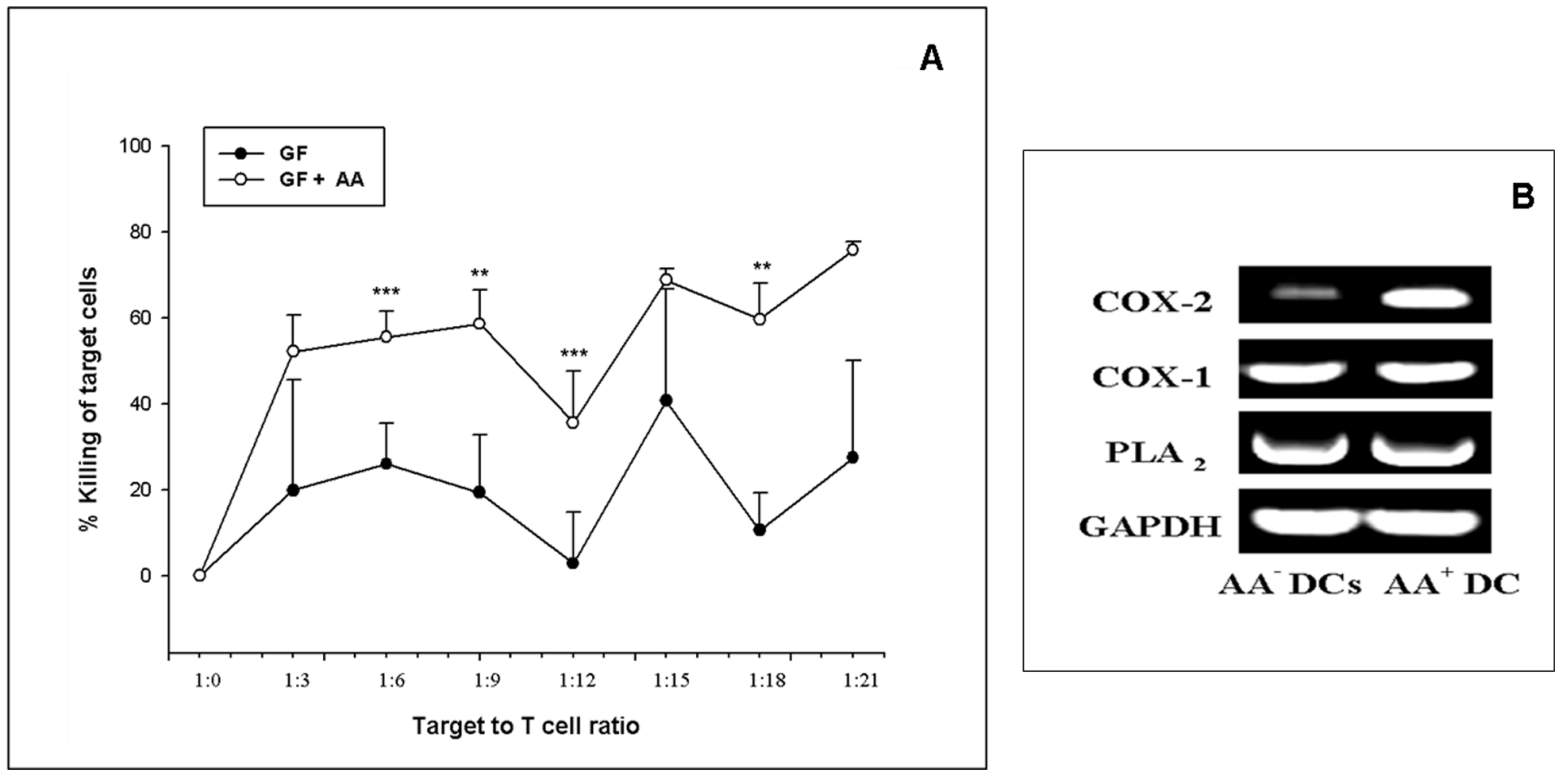
CTL and Transcript levels of key enzymes in AA^+^ DCs and AA^-^ DCs. (A) CTL data of one representative sample i.e. killing of the target (MCF-7) cells by CTLs generated by AA^+^ DCs/AA^-^ DCs pulsed with MCF-7 cell lysate. CTLs derived from AA^+^ DCs exhibited an improved effector function i.e. enhanced overall killing as compared to CTLs derived from AA^-^ DCs. The killing is significantly higher at four ratios of effector target cell concentrations. (*P≤0.05, **P≤0.01, ***P≤0.001). (B) Gene expression profile of three key enzymes associated with AA pathway along with the housekeeping gene GAPDH. There is a substantial up-regulation of transcript level of key enzyme COX-2 in the DCs cultured in presence of AA.

Figure 5A depicts CTL data from one representative sample. It is clearly seen that AA^+^ DCs are more efficient in effector function of T cells i.e. bringing about killing of MCF-7 cells in the CTL assay as compared to AA^-^ DCs. The killing is significantly higher at four ratios of effector target cell concentrations. The other two samples also showed similar trend (Fig. S5). CTLs derived from AA^+^ DCs exhibited an improved effector function i.e. enhanced overall killing as compared to CTLs derived from AA^-^ DCs. For the negative control set, AA^+^ DCs and AA^-^ DCs sets were matured with LPS, TNF-α and CD40L [20]. The CTLs thus generated were not effective in killing the target MCF-7 cell line. No killing was seen in either of these sets (data not shown). As an additional negative control for target specificity, CTLs obtained from MCF-7 lysate primed DCs were used in CTL assay against H1299 cell line but no killing effect was seen in these sets as well (CPM values for thymidine incorporation in case of control was 12649.33±30.73 SD. For pulsed and unpulsed group at highest target to T cell ratios were 12405.66±36.35 SD and 12537.33±27.47 SD respectively).

### The mRNA levels of key enzymes involved in AA metabolism are higher in AA^+^ DCs

The transcript levels of three enzymes associated with arachidonic acid pathway, such as COX-1, COX-2, PLA_2_, were detected by RT-PCR carried out as per standard methods. Gel images of RT-PCR are shown in Fig. 5B. There was a higher expression of COX-2 in the AA^+^ DCs set as compared to AA^-^ DCs set. Increased mRNA levels are indicative of active metabolism of the exogenously added AA in the test sets. However the differences in the expression of COX-1 and PLA2 between control and test sets were marginal. Taken together the *in vitro* data indicates that, AA^+^ DCs exhibited improved functional properties compared to AA^-^ DCs. Thus, though addition of AA does not alter the morphology and phenotype of DCs, the functions of these DCs are enhanced.

### Enhanced homing of AA^+^ DCs to bone marrow in NOD SCID mice

**Figure 6:**
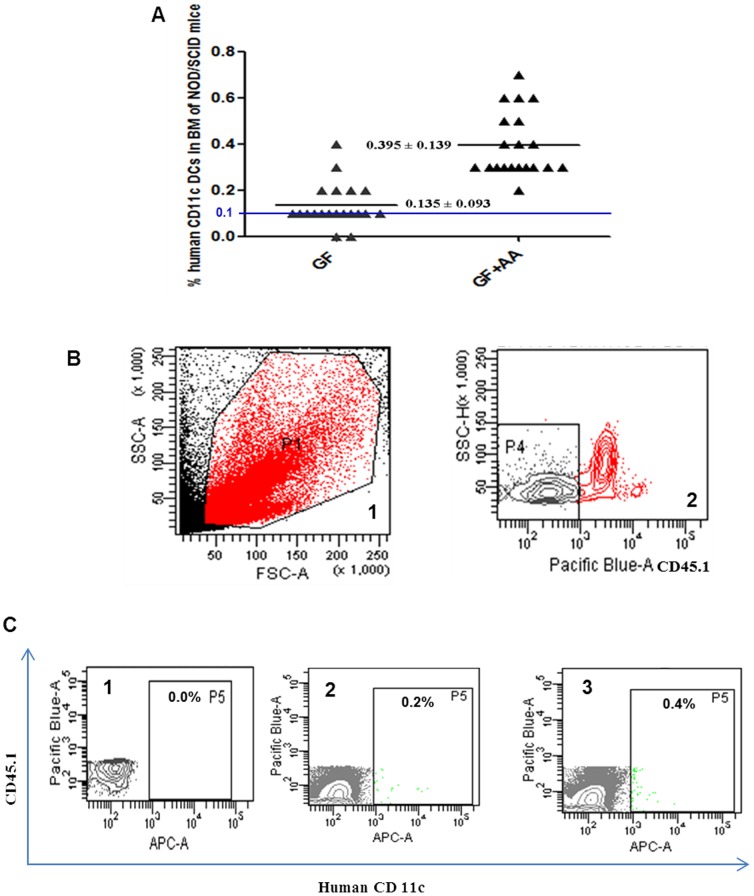
*In vivo* homing of the human AA^+^ DCs and AA^-^ DCs in NOD/SCID mice. (A) Scatter plot showing the percentage of DCs positive for human CD11c in bone marrow of individual mouse (n = 40), 18 hrs post-infusion. Significantly higher numbers of DCs were detected in BM of mice receiving AA^+^ DCs compared with mice receiving AA^-^ DCs (P≤0.001). Mice showing ≥0.1% human cells were considered positive for engraftment (Shown by a blue line). The bar indicates mean values with ± SD. (B) Representative Flow cytometry profile showing gating strategy 1) FSC/SSC, P1 gated cells were analyzes for mCD45.1 positive cells. 2) P4 represents gate for cells negative for mCD45.1, hCD11c were analyzed in P4 gate. (C) Representative Flow cytometry profile showing hCD11c positive cells in P5 gate of mice infused with 1) PBS, 2) AA^-^ DCs and 3) AA^+^ DCs.[Pacific blue- mCD45.1, APC- hCD11c].

To evaluate the *in vivo* homing ability of the *in vitro* generated DCs, we intravenously administered the cultured control and test DCs into sub lethally irradiated NOD/SCID mice as described in methods. 10^6^ AA^+^ DCs (test) and AA^-^ DCs (control) were used for intravenous infusion. The animals were sacrificed after 18 hr and the ability of human DCs to home was assessed by staining the BM cells with anti hCD11c and anti murine CD45.1 mAb. Intravenous injected DCs have been reported to home to bone marrow [24]. The percent homing in each set was compared to the PBS infused irradiated recipient to detect the background staining if any. It was observed that, the animals, which received the AA^+^ DCs, showed a higher migration in BM than the animals that received the AA^-^ DCs generated with growth factors alone. This correlated well with the *in vitro* results. Fig. 6A shows that significantly higher number of AA^+^ DCs migrated to BM of individual animals as compared to AA^-^ DCs (P≤0.001). The representative dot and contour plots showing the gating strategy are exemplified in Fig. 6B. Expression of hCD11c in one representative mouse infused with PBS, AA^-^ DCs and AA^+^ DCs is shown in Fig. 6C 1, 2 and 3 respectively. Thus the presence of AA in the culture was found to be beneficial in generating the DCs with a higher migration capacity.

## Discussion

DCs derived from UCB samples can serve as an allogeneic source of DCs for their potential use in cancer immunotherapy. Our published method leads to enrichment of a homogenous population i.e. myeloid interstitial DC subset [17], [20]. Here we demonstrated for the first time that the addition of AA at the differentiation step of our culture method showed beneficial effects thus further improving the quality of DCs generated from cord blood. The maturation status plays a decisive role in antigen presentation, costimulation and ultimately adjudicates whether the outcome is immunogenic or tolerogenic. DCs used in cancer immunotherapy should have strong immunogenic response. DCs exhibit their anticancer effect by capturing the tumor antigens [7], [8], [9]. The AA^+^ DCs showed higher antigen uptake compared to AA^-^ DCs underscoring the beneficial effect of AA addition to the cultures. Another important feature of DCs is their capacity to stimulate the proliferation of T lymphocytes in an allogeneic MLR and to generate effector CTLs. The inflammatory nature of many cancers creates an immunosuppressive environment that leads to suppression of DC-instructed effector CD4+ and CD8+ T cell responses [6]. The enhanced MLR in the culture system with AA may be attributed to production of more mature DCs which may help to cope up with immunosuppressive environment of cancer seen in the *in vivo* situation.

The migratory properties of DCs are of fundamental importance for their function and have been extensively investigated [25], [26]. DCs travel from bone marrow to the various tissues and from there to secondary lymphoid organs [1]. Our *in vitro* as well as *in vivo* data showed that DCs grown in presence of AA exhibited enhanced migration to CCL19 (*in vitro*) and to BM (*in vivo*). Michael A. Morse *et al.* demonstrated that DCs injected I.V, initially localize in the lungs and then redistribute to the liver, spleen, and bone marrow, but apparently not to the lymph nodes or tumor masses [24]. We had not observed any differences in migration of AA^-^, AA+ DCs to the spleen.We had selected 18 hrs post infusion as the time point in homing experiment. Studying the homing at different time points probably may give a different picture. CCR-7 is selectively expressed on mature DCs. The CCR-7 receptor and CCL-19 ligand interaction facilitate migration of mature DCs [26], [27]. Our data established the specificity of this interaction. Our results are in agreement with the findings of van Helden *et al.* where they report more efficient *in vitro* migration to CCL19 but no change in CCR7 expression on DCs after addition of PGE2 [28]. They attribute increase in migration to dissolution of podosomes by PGE2 as well as up regulation of MMP9 which is also essential for DC chemotaxis *in vitro* and *in vivo* migration. Whether the same mechanism operates in our system needs to be looked into.

DCs generated for immunotherapy purpose should have cytokine profile which supports the Th1 type of response. In other words, secretion of low levels of IL-10 and high levels of IL-12 is a desirable character for the DCs to be used for vaccination regimen. In our culture system AA addition results in improved IL12/IL10 ratio thus further improving their antitumor ability. Pawel Kalinski *et al.* [29] have shown that though PGE2 is reported as a suppressive inflammatory factor, it also contributes to the initiation of primary immune responses by facilitating the cytokine-induced final maturation and the increase in immunostimulatory capacity of DC, confirming the role of PGE2 as a Th2-promoting factor, acting at the APC level. Enzymes Cyclooxygenases (COX-1 and COX-2) convert the AA released by cPLA2 to PG endoperoxide H2, which is the precursor of series 2 prostanoids such as PGD2 and PGE2. Unlike COX-1, COX-2 is an inducible enzyme involved in the sustained production of prostanoids by many cell types [30]. Notably, COX-2 activity is necessary for strong Ab response following vaccination; especially when vaccines are poorly immunogenic or the target population is poorly responsive to immunization [31]. Thus the enhanced expression of COX-2 mRNA as seen in AA^+^ DCs may be favorable for their use of as anticancer agents. However one wonders which of the following metabolites of AA downstream pathway like LTB4, cysteinyl leukotrienes, 12–15-hetes, PGE2, and PGD2 are actually responsible for the beneficial effect. In future we propose to address these issues of delineating the pathway by appropriate use of pharmacological inhibitors.

Many DC-based clinical trials for cancer treatment have shown its safety and feasibility [9], [30]. The clinical efficacy of this therapy still needs to be improvised. Advances in biology lead to frequent improvements in the vaccine production protocols and therapeutics [32], [33], [34], [35], [36], [37]. Recent reports also illustrate that there is emerging evidence that PGE2 plays crucial roles in reciprocal crosstalk between dendritic cells and natural killer cell biology. Several NK cell functions (lysis, migration, proliferation, cytokine production) are influenced by PGE2, accentuating the role of PGE2 on DC– NK cell crosstalk and its subsequent impact on immune regulations in normal and immunopathological processes. [38] Some of the previous reports show that *in vitro* generated DCs are less efficient in migration and other functional activities [6], [19], [24] due to use of the cytokine IL-4 in the protocols [18]. IL-4 is known to adversely affect the AA metabolism. So we hypothesized that exogenous addition of AA in the culture medium may improve the functional activities of DCs. As per our expectation, AA^+^ DCs exhibited a better *in vitro* chemotaxis, T cell stimulation, CTL activity, Th1 favorable cytokine profile, antigen uptake and *in vivo* migration. *In vitro* manipulation of cellular vaccines is crucial for their successful use in clinics and thus our methodology, though it shows an incremental increase in the output, may add a new dimension in improvising the production of a potent immunotherapeutic agent. In other words these findings will be helpful in the better contriving of DC based vaccines for cancer immunotherapy with enhanced functionality.

## Supporting Information

Figure S1 **Flow chart depicting the design of the experiment.**
(TIF)

Figure S2 **Sequence of four primers used in the study.**
(TIF)

Figure S3 **AA^+^ DCs and AA^-^ DCs show similar morphology.** Phase contrast images of (A) Immature adhered DCs. (B) Both cultures show the typical veiled clusters of mature DCs. (C) Wright-Giemsa images of adherent cells show typical DC morphology. Original magnification for A is 10X, for B and C is 20X. The phase contrast images were cropped and enlarged.
(TIF)

Figure S4 **IL-12 p70/IL-10 ratio was higher in AA^+^ DCs as compared to AA^-^ DCs.** Concentrations of IL-12 p70 and IL-10 was in pg/ml.
(TIF)

Figure S5 **CTL assay of other two samples showing improved killing in AA^+^ DCs.**
(TIF)
