# Supplementary figures and images for: Exogenous Addition of Arachidonic Acid to the Culture Media Enhances the Functionality of Dendritic Cells for Their Possible Use in Cancer Immunotherapy
Source: PLoS One. 2014 Nov 4;9(11):e111759. doi: 10.1371/journal.pone.0111759 (PMC4219773; doi:10.1371/journal.pone.0111759)

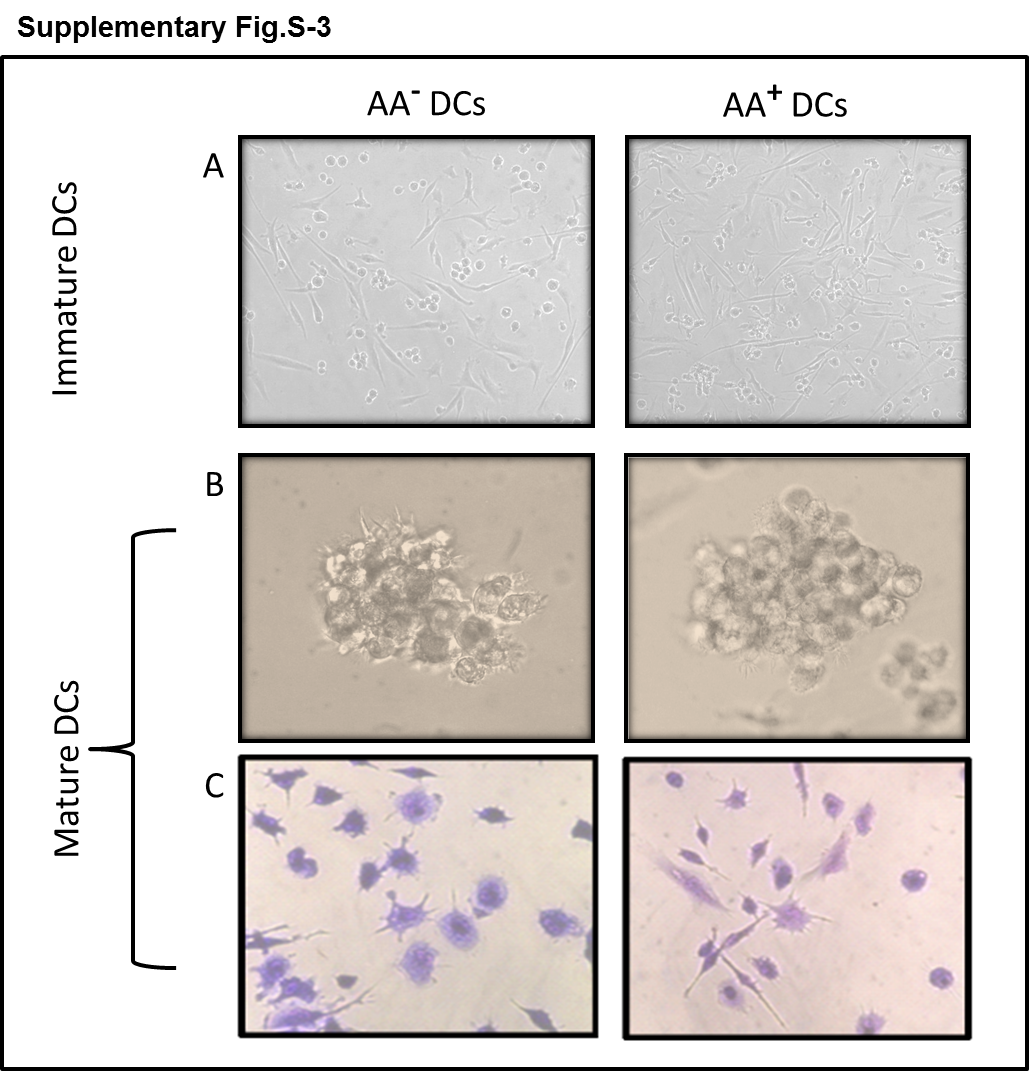

Supplement: Figure S3 — AA+ DCs and AA- DCs show similar morphology. Phase contrast images of (A) Immature adhered DCs. (B) Both cultures show the typical veiled clusters of mature DCs. (C) Wright-Giemsa images of adherent cells show typical DC morphology. Original magnification for A is 10X, for B and C is 20X. The phase contrast images were cropped and enlarged. (TIF) [file pone.0111759.s003.tif]

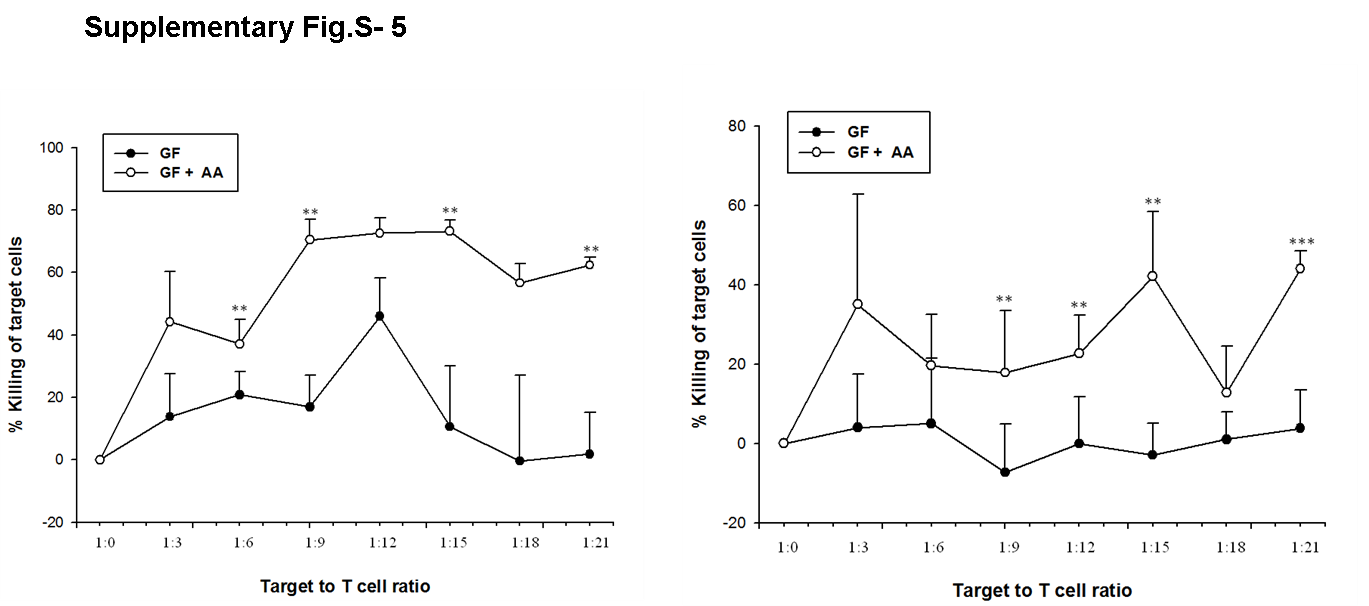

Supplement: Figure S5 — CTL assay of other two samples showing improved killing in AA+ DCs. (TIF) [file pone.0111759.s005.tif]
